# Sharing of Antimicrobial Resistance Genes between Humans and Food Animals

**DOI:** 10.1128/msystems.00775-22

**Published:** 2022-10-11

**Authors:** Huiluo Cao, Salim Bougouffa, Tae-Jin Park, Andes Lau, Man-Ki Tong, Kin-Hung Chow, Pak-Leung Ho

**Affiliations:** a Carol Yu Center for Infection and Department of Microbiology, University of Hong Kong, Hong Kong, People’s Republic of China; b Computational Bioscience Research Center and Bioscience Core Lab, King Abdullah University of Science and Technology, Thuwal, Saudi Arabia; c HME Healthcare Co., Ltd., Suwon-si, Gyeonggi-do, Republic of Korea; d Department of Microbiology, Queen Mary Hospital, Hong Kong, People’s Republic of China; California State University, Stanislaus

**Keywords:** antibiotic resistance gene, food animals, human gut, metagenome, mobile genetic element

## Abstract

The prevalence and propagation of antimicrobial resistance (AMR) are serious global public health concerns. The large and the ever-increasing use of antibiotics in livestock is also considered a great concern. The extent of the similarity of acquired antibiotic resistance genes (ARGs) between humans and food animals and the driving factors underlying AMR transfer between them are not clear, although a link between ARGs in both hosts was proposed. To address this question, with swine and chicken as examples of food animals, we analyzed over 1,000 gut metagenomes of humans and food animals from over the world. A relatively high abundance and diversity of ARGs were observed in swine compared with those in humans as a whole. Commensal bacteria, particularly species from *Clostridiales*, contribute the most ARGs associated with mobile genetic elements (MGEs) and were found in both humans and food animals. Further studies demonstrate that overrepresented MGEs, namely, Tn*4451*/Tn*4453* and Tn*As3*, are attributed mainly to the sharing between humans and food animals. A member of large resolvase family site-specific recombinases, TnpX, is found in Tn*4451*/Tn*4453* which facilitates the insertions of the transient circular molecule. Although the variance in the transferability of ARGs in humans is higher than that in swine, a higher average transferability was observed in swine than that in humans. In conclusion, the potential antibiotic resistance hot spots with higher transferability in food animals observed in the present study emphasize the importance of surveillance for emerging resistance threats before they spread.

**IMPORTANCE** Antimicrobial resistance (AMR) has proven to be a global public health concern. To conquer this increasingly worrying trend, an overarching, One Health approach has been used that brings together different sectors, but the fundamental knowledge of the relationship between humans, food animals, and their environments is not mature yet or is lacking in some aspect. With swine and chicken as examples of food animals, a large global data set of over 1,000 human and food animal gut metagenomes was analyzed with a focus on acquired antibiotic resistance genes (ARGs) associated with mobile genetic elements (MGEs) to answer this question. Outputs from this work open a new avenue to further our understanding of ARG transferability in food animals. It is a necessary milestone to better equip governmental agencies to monitor and pre-empt antibiotic resistance hot spots. This work will assist and give guidance on how to decipher other links within any One Health initiatives with expected positive feedback to human health.

## INTRODUCTION

As a serious global public health concern, antimicrobial resistance has been aggravated by the increasing incidence of multidrug resistance in clinical pathogens (1). Genes conferring antimicrobial resistance (AMR) in pathogens are syntenic with mobile genetic elements (MGEs), such as integrons, transposons, plasmids, and prophages, suggesting that they may be disseminated within a mobile multidrug resistance cassette from commensal bacteria to pathogens. Livestock is considered a major source of acquired antibiotic resistance genes (ARGs) (2), especially in low- and middle-income countries (3). Bacteria in animals that are treated with antibiotics can develop antibiotic resistance, and these bacteria, which might carry resistance genes, then can be transmitted from animals to humans and vice versa. This intertransmission of ARGs can occur through food, by direct contact between humans and animals, or through shared environmental resources, such as contaminated water (4). This information emphasizes the importance of the continued prioritization and the surveillance of the antibiotic resistome, particularly in hot spots where there is a high likelihood of resistance gene evolution and transfer between bacterial hosts.

Clinically significant ARGs, such as New Delhi metallo-beta-lactamase (*bla*_NDM_) and plasmid-borne colistin-resistance genes (*mcr-1*, colistin that is the last resort antibiotic for human health) which are found in common pathogens, have been studied broadly and were observed simultaneously in food animals and humans, indicating potential links between these two types of hosts (5, 6). *mcr-1* was found initially through an active surveillance program in China that monitored ARGs in food animals (7), and a follow-up study on the global distribution posited a possible origin of *mcr-1* in Chinese livestock (8) indicating the potential zoonotic origin of *mcr-1* in humans. These studies suggest that food animals could be an important source of clinically significant ARGs in human populations. However, the extent that ARGs occur in commensal bacteria, which have been proven to transfer to pathogens and are shared by humans and food animals, is undetermined. Monitoring of the resistome in humans and food animals could provide more solid evidence and a comprehensive understanding of the migration of ARGs.

Indeed, the large and expanding use of antibiotics in livestock, particularly in China, is of a great concern in light of the threat of antibiotic resistance (3, 9). In Hong Kong, 80% of the food animals are imported from mainland China (10), ARGs in imported food animals from China are thought to have strong influence on ARGs in Hong Kong populations, and this influence has also been observed in other countries, such as European countries (3). In this work, we exploit swine and chicken as examples to gauge the sharing and the transferability of ARGs with humans on a global scale. The transferable capability of ARGs was evaluated through the corresponding mobile genetic cassette identified in both types of hosts and by the taxonomy of bacteria.

## RESULTS

### Diversity and relative abundance of ARGs.

**FIG 1:**
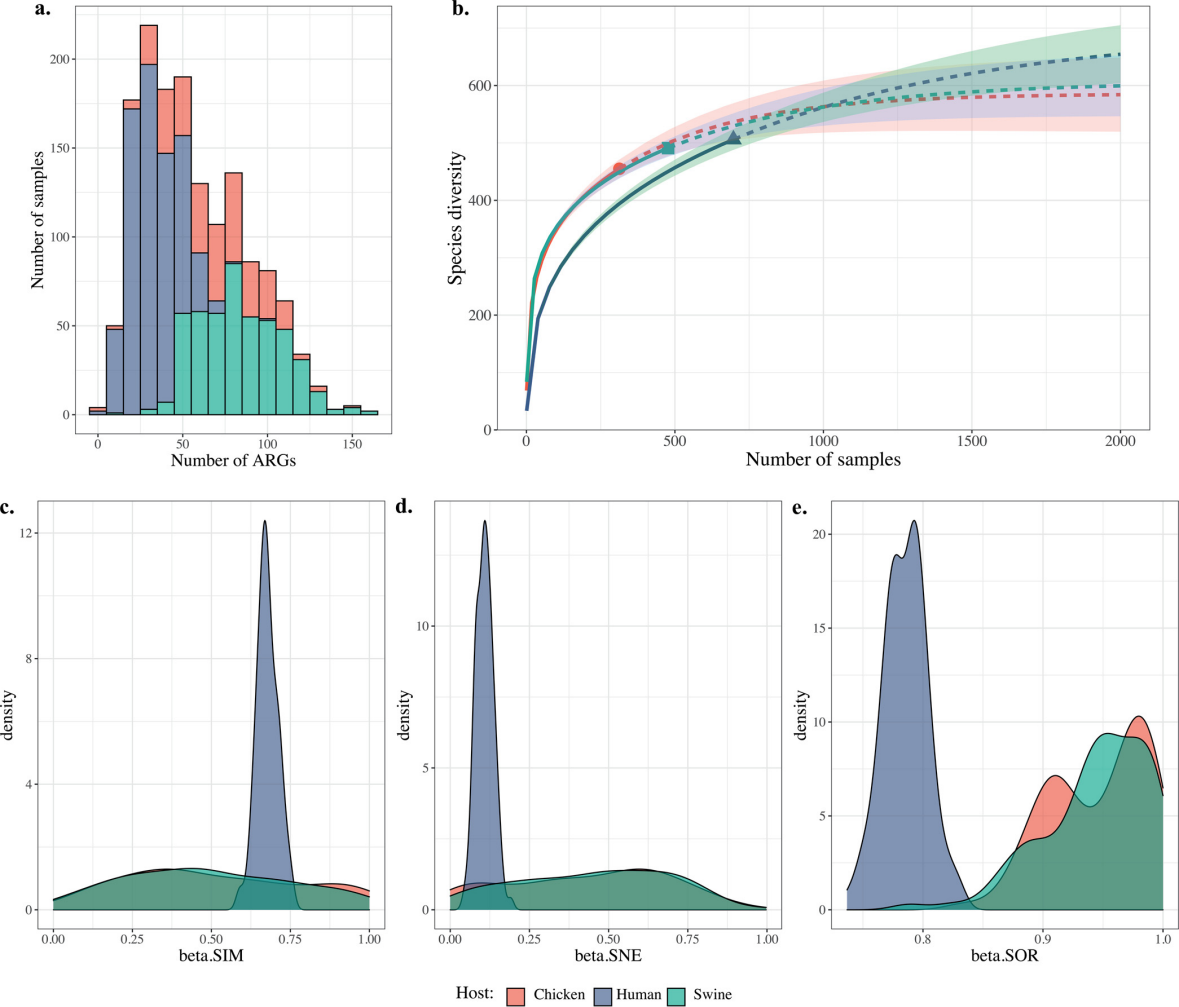
Diversity comparison of acquired ARGs in the hosts human, chicken, and swine. (a) The frequency distribution of ARGs in all samples; *x* axis shows bins with 10. (b) Rarefaction curve of the numbers of ARGs interpolated and extrapolated for both types of hosts that are calculated by iNEXT and plotted using R. (c to e) Distribution of 3 Sørensen-based multiple-site dissimilarities accounting for the spatial turnover and the nestedness components of beta diversity, and the sum of both values for ARGs in both types of hosts were calculated using R package betapart with 100 resample times for 10 samples. beta.SIM, value of the overall beta diversity, measured as Sorensen dissimilarity; beta.SOR, value of the turnover component, measured as Simpson dissimilarity; beta.SNE, value of the nestedness component, measured as nestedness-resultant fraction of Sorensen dissimilarity. The same color pattern was used for all panels to represent the host type.

In present study, a total of 1,487 gut metagenomes of humans and food animals from globally geographical locations, including our recently sequenced 21 human gut microbiota samples, were collated and analyzed (see Fig. S1 in the supplemental material; Table 1; see Table S1 online at https://doi.org/10.6084/m9.figshare.20514555.v1). The numbers of observed ARGs in most human samples were lower than those from food animals, including swine and chicken (Fig. 1a), but the distributions of ARGs of sequencing reads in both types of hosts were comparable (see Fig. S2 in the supplemental material). Rarefaction curves indicated the nonsaturation of samplings for samples from both hosts (Fig. 1b; see Fig. S3 in the supplemental material), and therefore, additional sequencing may be needed in the future, especially from those low- and middle-income countries for which data are not available currently. All three beta diversity indices exhibit greater differences within human samples than within food animal samples (Fig. 1c to e).

**TABLE 1:** Numbers of metagenomes in each region and host in the present study

Region	No. of metagenomes by host		
	Human	Swine	Chicken
Hong Kong	74	0	0
China	85	97	135
France	12	120	20
Denmark	55	120	20
Netherlands	0	20	20
Germany	62	20	19
Spain	51	20	20
Belgium	0	20	20
Italy	52	20	20
Poland	0	20	20
Bulgaria	0	21	19
Japan	31	0	0
Austria	16	0	0
Sweden	39	0	0
Canada	18	0	0
Peru	15	0	0
Salvador	15	0	0
USA	61	0	0
India	110	0	0
Total	696	478	313

10.1128/msystems.00775-22.1 FIG S1 Samples selected in the present study. The numbers of samples used from each region are shown in semicircles, with two types of hosts, namely, human and food animals, in different colors. The detailed information of samples is in Table S1. Download FIG S1, TIF file, 2.6 MB. Copyright © 2022 Cao et al. 2022 Cao et al. https://creativecommons.org/licenses/by/4.0/ This content is distributed under the terms of the Creative Commons Attribution 4.0 International license.

10.1128/msystems.00775-22.2 FIG S2 The distributions of ARGs per Gb reads in both types of hosts, which is calculated based on Table S1. Download FIG S2, TIF file, 0.9 MB. Copyright © 2022 Cao et al. 2022 Cao et al. https://creativecommons.org/licenses/by/4.0/ This content is distributed under the terms of the Creative Commons Attribution 4.0 International license.

10.1128/msystems.00775-22.3 FIG S3 Rarefaction curves of ARGs in hosts of human and food animals as a whole. Rarefaction curve of the numbers of ARGs interpolated and extrapolated for both types of hosts that were calculated by iNEXT and plotted using R. Download FIG S3, TIF file, 2.0 MB. Copyright © 2022 Cao et al. 2022 Cao et al. https://creativecommons.org/licenses/by/4.0/ This content is distributed under the terms of the Creative Commons Attribution 4.0 International license.

**FIG 2:**
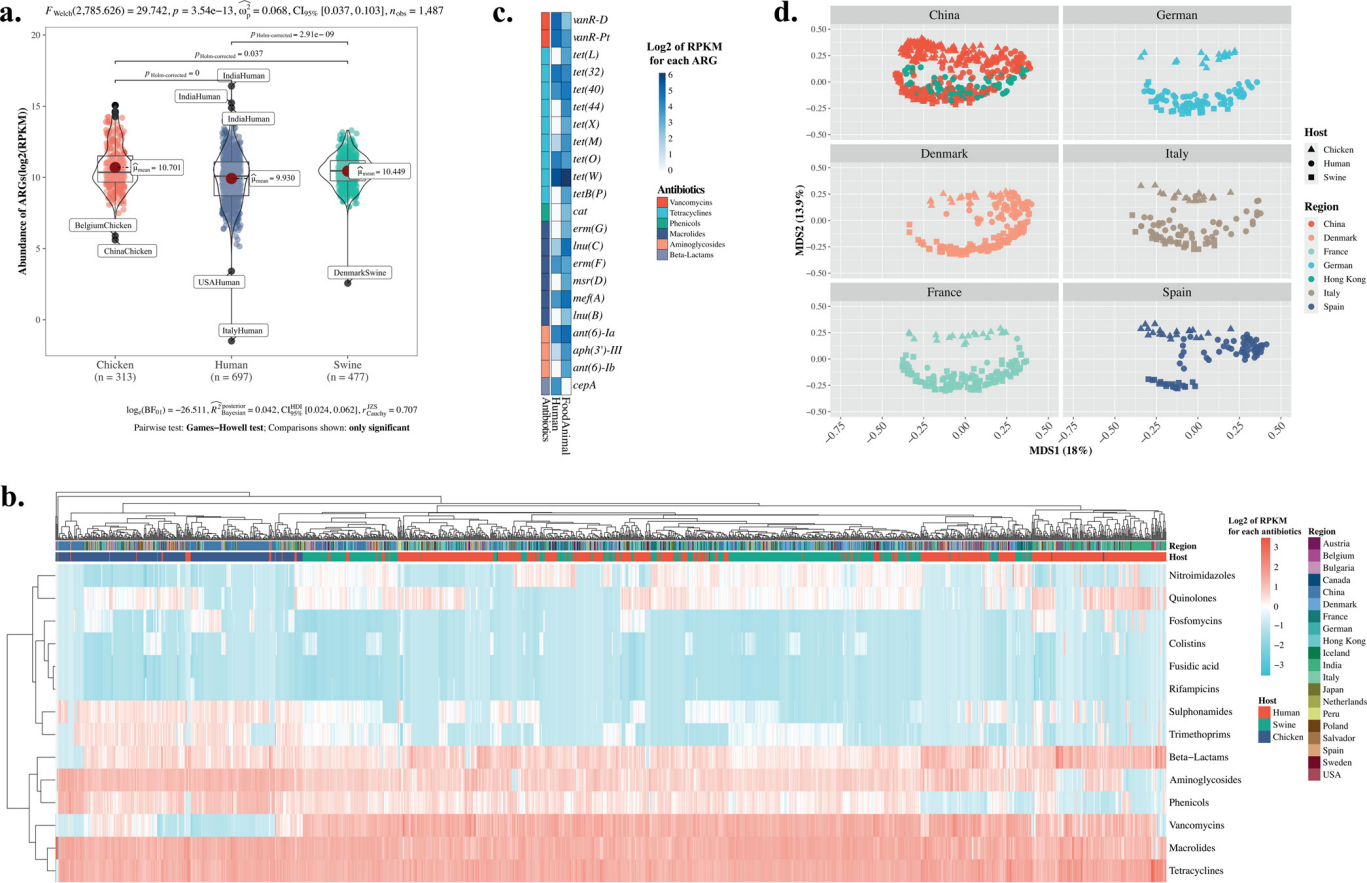
Comparison for the relative abundance of acquired ARGs in human and food animals. (a) Relative abundance of summed ARGs in two types of human and food animal hosts, and each colored point represents one sample. The difference between two types of groups was compared using Wilcoxon rank-sum test with a *P* value of <0.05. (b) Heatmaps of the relative abundance of acquired ARGs in human and food animal fecal samples in the present study clustered in each group of hosts in columns and class of ARGs in rows. Relative abundance of each ARG has been standardized with base 2 logarithm and scaled as shown in the scale bar on the top right of the figure. All labels of groups are in Table 1 and Table S1. Assignations of ARGs to antibiotics referred to WTO ATC code J01. (c) Heatmap of significantly different relative abundance of acquired ARGs (represented by log_2_ RPKM in each gene) in two types of hosts, namely, human and food animals, calculated using the Wilcoxon tank-sum test with false-discovery rate (FDR) corrections. Names of ARGs are adapted from the ResFinder database. (d) Principal coordinate analyses of the relative abundance of acquired ARGs for each pair of hosts of human and food animals in the same region. Since most pigs are imported from China in Hong Kong, human in Hong Kong and China and swine from China were plotted in the same faceted panel. Each colored point or shape represents one sample with shapes and colors to differentiate hosts.

The relative abundance of ARGs in swine is significantly higher than that in human hosts (Fig. 2a; see Table S2 online at https://doi.org/10.6084/m9.figshare.20514555.v1). Considering ARGs per host type and by sampling region, the highest abundance and the highest diversity (both Shannon and Simpson indices) of ARGs were observed in swine from China (pairwise Wilcoxon signed-rank test with Benjamini-Hochberg method to correct, *P < *0.05) (see Fig. S4 in the supplemental material). Within the human cohorts, the Indian population, surprisingly, has the highest relative abundance of ARGs, as supported by the pairwise Wilcoxon signed-rank test (corrected with the Benjamini-Hochberg method, *P < *0.05) (Fig. S4a). As for humans versus food animals, we detected a higher abundance of ARGs in food animals than that in humans in all countries except France and Italy (Fig. S4b).

10.1128/msystems.00775-22.4 FIG S4 Relative abundance and diversity of acquired ARGs in human and food animal hosts. (a) Relative abundance and diversity of summed acquired ARGs in each group of human and food animal hosts. The relative abundance was calculated by log_2_ values with reads per kilobase of gene and million reads (RPKM). (b) Abundance and diversity indices (Shannon and Simpson indices) of ARGs in all types of hosts. All these indices were calculated using R scripts based on the relative abundance data of all ARGs in each sample. Download FIG S4, TIF file, 2.8 MB. Copyright © 2022 Cao et al. 2022 Cao et al. https://creativecommons.org/licenses/by/4.0/ This content is distributed under the terms of the Creative Commons Attribution 4.0 International license.

We clustered all ARGs by class in both groups of hosts into a heatmap to show those ARG classes that are significantly abundant (Fig. 2b). ARGs conferring resistance to tetracyclines, vancomycin, and macrolides are observed widely in most samples, while those to quinolones, phenicols, and aminoglycosides show large differences in different hosts. For details, refer to Fig. 2c where we highlight ARGs that are statistically different in the relative abundance of ARGs within both types of hosts. All ARGs but *cepA*, which encodes a class A beta-lactamase, are more abundant in food animals than in humans (Fig. 2c). In particular, a few ARGs, for instance, tetracycline resistance genes, exhibit high abundance in both types of hosts. To obtain more details of shared ARGs between different hosts in each region, heatmaps of ARGs for each class of antibiotic were plotted (see Fig. S8 online at https://doi.org/10.6084/m9.figshare.20522313.v2). A relatively higher abundance of ARGs against most antibiotic classes were observed in Indian human and Chinese chicken samples, particularly for beta-lactams and ARGs conferring resistance to quinolones. Variations of the relative abundance of ARGs within the same class of antibiotic were observed. Samples from Chinese swine were enriched for ARGs conferring resistance to phenicols, aminoglycosides, and tetracyclines but lack ARGs related to quinolone (Fig. 2b; see Fig. S8 at online https://doi.org/10.6084/m9.figshare.20522313.v2). All these results do not show the possible sharing between two types of hosts. Thus, in the following section, the detailed shared ARGs between two types of hosts are described.

The principal coordinate analysis separated ARGs in food animals from those in human hosts for all but a few samples (see Fig. S5 in the supplemental material). The separation of ARGs by host sources is consistent across all regions. But the separation distance between host types varies with Spain and Denmark having the most prominent separation while France had the least prominent separation (Fig. 2d). The human gut resistome is closer to that in swine than that in chicken (Fig. 2d). This finding is consistent with observations in Fig. S5. For these separations between hosts, differences in antibiotic exposure might be an underlying reason. Unexpectedly, there is a closer distance between ARGs from human hosts in Hong Kong to swine hosts in China than to human hosts in China (Fig. 2d). This finding drives us to explore the reasons in the following section since it indicates the potential risk of ARG transfer between two types of hosts.

10.1128/msystems.00775-22.5 FIG S5 Principal coordinate analysis of the relative abundance of acquired ARGs in each group of fecal samples. It was based on genus-level Bray-Curtis dissimilarity using the *vegan* package in R with contours showing. Download FIG S5, TIF file, 2.4 MB. Copyright © 2022 Cao et al. 2022 Cao et al. https://creativecommons.org/licenses/by/4.0/ This content is distributed under the terms of the Creative Commons Attribution 4.0 International license.

### Sharing of ARGs between humans and food animals.

**FIG 3:**
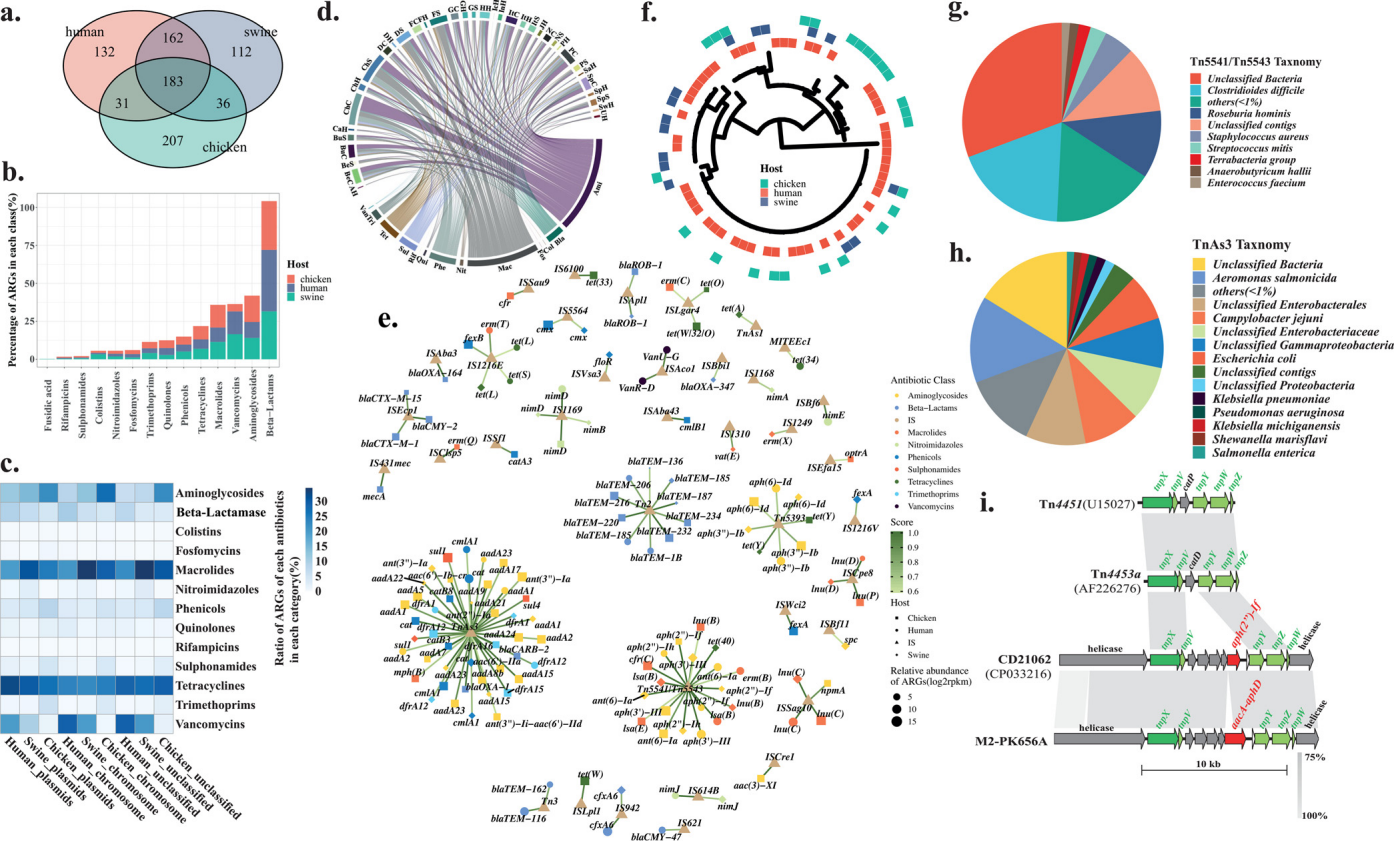
Shared ARGs in humans and food animals. (a) The Venn plot of the numbers of ARGs shared by human and food animals in total. (b) Frequency of ARGs assigned to each antibiotic class in both types of hosts. (c) Heatmap of the frequencies of ARGs assigned to each antibiotic class predicted to occur in plasmids and the chromosome in both types of hosts. (d) Circos diagram shows the presence of the shared antibiotic classes conferred by ARGs associated with mobile genetic elements in both food animals and human hosts (183 ARGs). Links represent the frequencies of ARGs observed in each type of samples. Abbreviations for each type of samples can be found in Table S1; antibiotics classes and their abbreviations are as follows: vancomycin, Van; trimethoprim, Tri; tetracycline, Tet; sulfonamide, Sul; rifampicin, Rif; quinolone, Qui; phinecol, Phe; nitroimidazole, Nit; macrolide, Mac; fosfomycin, Fos; colistin, Col; beta-lactams, Bla; and aminoglycoside, Ami. (e) Network of representative ARGs shared by both types of hosts and their associated insertion sequences. The size of each shape represents the relative abundance of ARGs. The color of each shape indicates the class of antibiotic to which ARGs are resistant. The edges connected by nodes are scores that strand for the ratio of each ARGs within all ARGs associated with the same IS. (f) Phylogenetic trees of representative ARG, *ant(6)-Ia*, that is associated with the same IS, Tn*4451*/Tn*4453*, in both types of hosts, namely, human (red) and food animal (chicken [green] and swine [blue]). Taxonomies of contigs possessing two major groups of ISs, namely, Tn*4451*/Tn*4453* (g) and Tn*As3* (h) that are associated with ARGs. (i) The alignment of the representative Tn*4451*/Tn*4453*-like transposons (CD21062 and one human sample, namely, M2-PK656A from Hong Kong) with typical Tn*4451*/Tn*4453*.

In total, 863 acquired ARGs with different variants from all microbiota were observed in the present study, of which 493 were identified to be shared by all swine gut microbiota, while 457 and 508 ARGs were observed in all chicken and human gut microbiota, respectively (Fig. 3a). Among these 863 ARGs, 345 ARGs were shared by humans and swine and 214 were shared by humans and chicken (Fig. 3a). Except for ARGs conferring resistance to fusidic acid that are absent in human hosts, the frequencies of the occurrence for all classes of ARGs in both types of hosts were comparable (Fig. 3b). When both types of hosts were sampled in the same regions, 303 ARGs were observed in both human and swine hosts (see Tables S3 and S4 online at https://doi.org/10.6084/m9.figshare.20514555.v1).

Since mobile genetic elements are the main contributors for the transferability of ARGs, information about ARGs located on plasmids or chromosomes was determined using contigs they inhabited. Although we have used several methods to classify the contigs into chromosomes, 21.4% of contigs carrying ARGs (21,761 out of 101,945) are unclassified. In total, the highest ratio of plasmids versus chromosomes was observed in chicken, and it was lowest in human (chicken, 2.3 [17,942/7,801]; swine, 1.1 [17,364/15,990]; humans, 0.84 [9,635/11,452]). Within each type of contig, the ratio of contigs carrying ARGs conferring resistance to each class of antibiotics demonstrates that ARGs conferring resistance to aminoglycosides, macrolide, tetracycline, and vancomycin are major classes (Fig. 3c).

To further explore the potential transferability of these ARGs between the two types of hosts, insertion sequence (IS) elements were identified in all contigs carrying ARGs in all samples. Taxonomic assignments of these contigs and whether these contigs are located on a chromosome or on plasmids were determined. The main groups of ARGs associated with ISs in each type of samples were plotted (Fig. 3d). Aminoglycoside- and macrolide-related ARGs are abundant in both types of hosts. ARGs conferring resistance to phenicols were observed mainly in swine from China. The main ISs associated with all classes of ARGs were identified and plotted in a network which shows two major clusters (Fig. 3e). Tn*4451*/Tn*4453* is the hub of ARGs conferring resistance to aminoglycosides and macrolides that were observed in both types of hosts (Fig. 3e). Another type of transposon, namely, Tn*As3*, was observed as the hub of aminoglycoside, phenicol, and trimethoprim resistance genes. Both types of hosts are also observed to have these ARGs. To confirm the observation that both types of hosts carry the same ARG associated with the same IS, one phylogenetic tree was constructed using the *ant(6)-Ia* gene that is connected between two types of hosts through Tn*4451*/Tn*4453* (Fig. 3f). Additionally, 10 ARGs with clinical significance were also clustered and plotted in the network to show the connections of these ARGs in different types of hosts (see Fig. S9 online at https://doi.org/10.6084/m9.figshare.20522313.v2), and one representative ARG, namely, *aac(3)*, was chosen to be used in the phylogenetic analysis (see Fig. S6 in the supplemental material). The phylogenetic analysis indicates that several dominant variants contribute to the sharing. The contigs possessing this type of transposon were classified into taxonomic units demonstrating that several dominant groups of commensal bacteria possess these transposons with ARGs (Fig. 3g and h). The gene arrangement in Tn*4451*/Tn*4453* was deciphered, and one key recombinase encoded by *tnpX* was observed (Fig. 3i). TnpX has been shown to play a major role in the excision of mobile elements (11). All the contigs carrying Tn*4451*/Tn*4453* were observed mainly in human samples from China and Hong Kong, while it also occurred in swine samples from other geographical regions (see Table S2 at https://doi.org/10.6084/m9.figshare.20514555.v1). In these Tn*4451*/Tn*4453*-carrying contigs, particularly, the recently reported ARG *cfr(C)* encoding resistance to oxazolidinones was observed.

10.1128/msystems.00775-22.6 FIG S6 Phylogenetic tree of *aac(3)* extracted from all samples. The phylogenetic tree was constructed using IQ-TREE, and information of the relative abundance of each sequence and the associated IS were mapped onto the phylogenetic tree as well. Download FIG S6, TIF file, 1.7 MB. Copyright © 2022 Cao et al. 2022 Cao et al. https://creativecommons.org/licenses/by/4.0/ This content is distributed under the terms of the Creative Commons Attribution 4.0 International license.

### Sharing of ARGs between human and swine in China and Hong Kong.

The observation of a close relationship of ARGs between humans and swine gut drove us to further study the ARGs between these two types of hosts by taking those from Hong Kong and China as examples. In total, 5,631 and 3,173 contigs carrying ARGs were identified in human samples from Hong Kong and China, respectively, compared with 13,969 contigs identified in swine of China (more details of these contigs, see Table S5 in the supplemental material and Fig. S8 online at https://doi.org/10.6084/m9.figshare.20514555.v1). Among these contigs, IS elements were predicted on 858 (15.2%), 458 (14.4%), and 1,832 (13.1%) in human samples from Hong Kong and China and swine samples in China, respectively.

**FIG 4:**
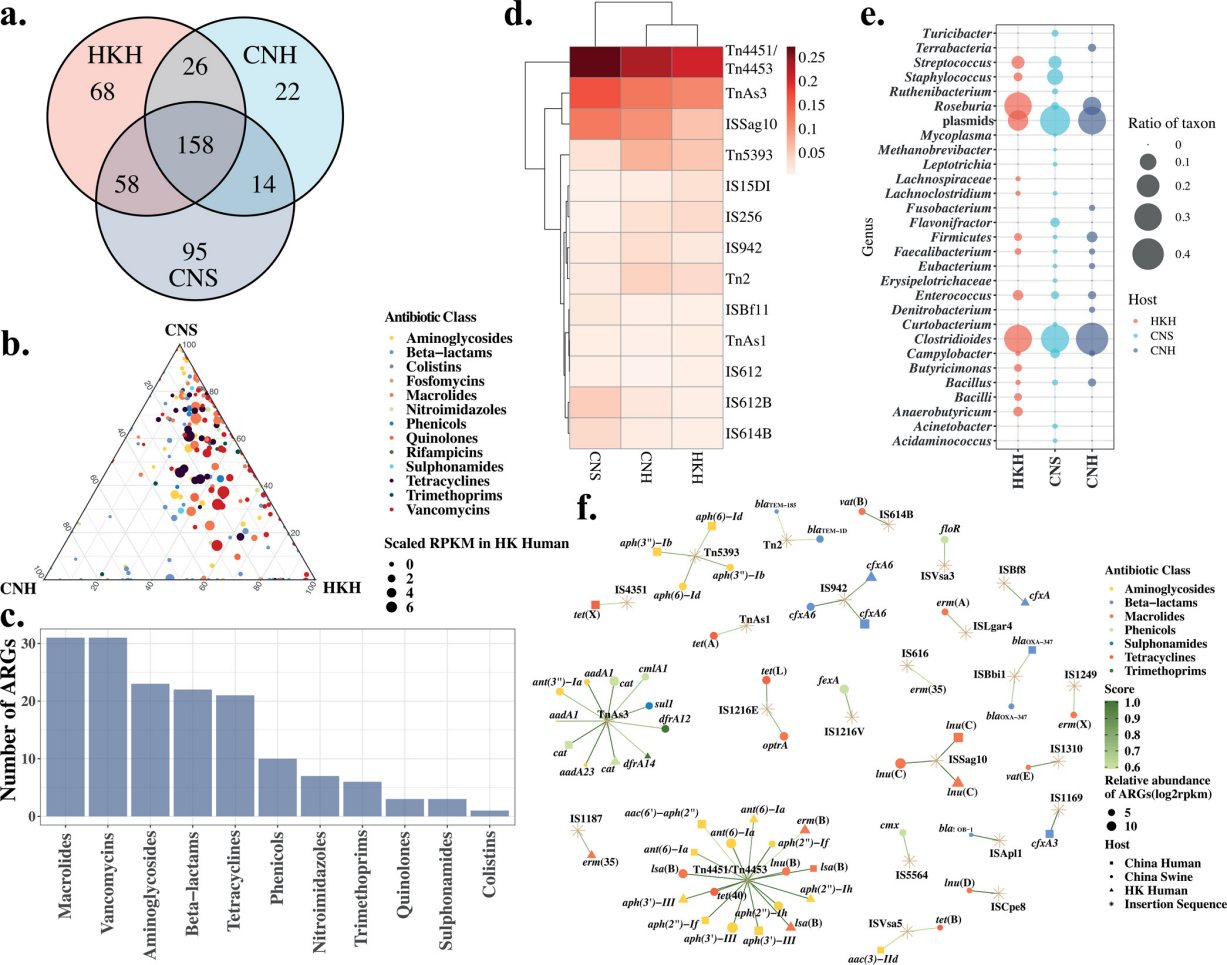
Shared antibiotic resistance genes between CNH, CNS, and HKH groups. (a) The Venn plot of the numbers of ARGs shared by pairs of host groups. (b) Ternary plot of shared ARGs for each pair of hosts. The size of the dot indicates the abundance of ARGs in human of Hong Kong. (c) The number of ARGs in each class shared by three groups of hosts (*n* = 158). (d) Heatmap of the frequencies of insertion elements associated with ARGs higher than 0.005 in all three groups of hosts. Two-ways clustering in column and row were performed. (e) The frequencies of taxonomic groups on the genera level that carry ARGs and insertion element Tn*4451*/Tn*4453* in all three groups of hosts. (f) Network of ARG-associated insertion sequences in three types of hosts. The size of the shape represents of the relative abundance of ARGs. The color stands for the class of antibiotic to which ARGs are resistant. The shape shows the type of hosts or insertion elements. The scores that represent the ratios of ARGs associated with ISs are illustrated as a color gradient of links between nodes. CNS, China swine; HKH, Hong Kong human; CNH, China human.

Results reveal that more ARGs (216 ARGs) in human guts from Hong Kong are shared with swine guts from China than those in other geographical regions (Fig. 4a; see Table S5 at https://doi.org/10.6084/m9.figshare.20514555.v1). Enrichment of *oqxAB* (encoding efflux pump), *fosA* (fosfomycin resistance gene), and *aph* (encoding aminoglycoside phosphotransferase) variants are ARGs shared among these three host groups. The relative abundance of shared ARGs in each group was shown in Fig. 4b. It is clearly demonstrated that more ARGs conferring resistance to aminoglycosides, macrolides, tetracyclines, and vancomycin are shared by China swine and Hong Kong human samples. When we compared Hong Kong human samples with China swine and human samples, 158 ARGs were shared by these three groups (Fig. 4a). The most abundant ARGs associated with ISs confer resistance to macrolides, followed by those assigned to vancomycin, aminoglycoside, beta-lactam, and tetracycline classes (Fig. 4c).

Mobile genetic elements, including IS, resolvase, phage integrase and recombinase, and conjugation elements were identified on the contigs possessing ARGs. The taxonomy of these contigs carrying ARGs was annotated using Kraken2. Contigs assigned to plasmids were identified as well. In all three groups of microbiomes, Tn*4451*/Tn*4453* was identified as the most common IS, as shown by only considering both types of hosts as a whole (Fig. 4d). Tn*4451*/Tn*4453* was observed to be occupied by different taxonomic groups, and it was correlated with diverse ARGs (Fig. 4e and f). In the Hong Kong human samples, *aph* genes conferring resistance to aminoglycosides were correlated with Tn*4451*/Tn*4453*. In this study, Tn*4451*/Tn*4453* was carried mainly by chromosomes from *Clostridioides*, *Roseburia*, and Streptococcus species and plasmids in the Hong Kong cohort (Fig. 4e). Interestingly, close strains of *Clostridioides* and *Roseburia* species carrying Tn*4451*/Tn*4453* were also observed in human and swine hosts of China (Fig. 4e). Further study demonstrates that Clostridioides difficile is the main host of Tn*4451*/Tn*4453* in three types of hosts in China and Hong Kong. Tn*As3* was observed mainly in *Enterobacteriaceae*, including in Escherichia coli, Klebsiella sp., and Salmonella sp., with more diverse ARGs. *Bacteroides* sp. with more variable insertion elements was observed in three groups of hosts.

Since the unique feature of the Hong Kong samples was observed, more details in ARGs were surveyed. In all Hong Kong samples, 310 ARGs were identified, but only one [*erm(B)*] was present in all subjects, compared with 17 to 97 ARGs (median, 46.5) observed in each sample. However, 30 ARGs were observed in half of the subjects from Hong Kong (*n* ≥ 37 out of 74), and 112 ARGs were unique in only one subject. Commensal bacteria, including *Clostridiales* (*Ruminococcus* and *Faecalibacterium*) and *Bacteroidales* (*Bacteroides*, *Alistipes*, and *Coprococcus*) species, were observed as the predominant group carrying IS-associated ARGs, but there was no obvious trend of distributions by populations, age, or sex (see Fig. S7 in the supplemental material). A batch of ARGs was observed with a higher abundance than the abundance of contigs that these ARGs were dwelling in, in particular, *tet(34)*, *erm(B)*, *bla_CTX-M_*, and *tet(Q)*. The associations of ARGs with bacterial hosts and IS elements are shown in Fig. S7. Tn*4451*/Tn*4453* was observed with more links with ARGs, particularly quinolone resistance genes in *Bacteroides* (Fig. S7). *Clostridioides* bacteria carry more ARGs assigned to vancomycin, macrolides, and quinolones. ARGs conferring resistance to beta-lactams were overrepresented in *Clostridioides*, *Eubacterium*, and plasmids.

10.1128/msystems.00775-22.7 FIG S7 Antibiotic resistance gene profiles in Hong Kong cohort. (a) frequencies of ARGs assigned to each class in the Hong Kong cohort. (b) Heatmap and clustering of assigned taxa of contigs, including ARGs associated with insertion elements in each subject. (c) Plot of the abundance (represented by RPKM) of ARGs associated with IS and the relative abundance (represented by coverage of mapped reads) of contigs. (d) Frequencies of ARGs associated with insertion elements in each subject classified into age groups (1, <18 years old; 2, >18 and <40; 3, >40 and <50; 4, >50 and <60). (e) Hive map representation showing all classes of ARGs associated with insertion elements and genera identified. Orange lines represent occurrence with over 100 counts, while blue lines represent occurrence lower than 100 counts. All samples from the present study are in red or grouped in the plot b (HKU_Yu from Yu et al [18]; HK_HKU from the present study). Download FIG S7, TIF file, 2.6 MB. Copyright © 2022 Cao et al. 2022 Cao et al. https://creativecommons.org/licenses/by/4.0/ This content is distributed under the terms of the Creative Commons Attribution 4.0 International license.

### Transferability of ARGs in human and swine hosts and main ARG contributors.

**FIG 5:**
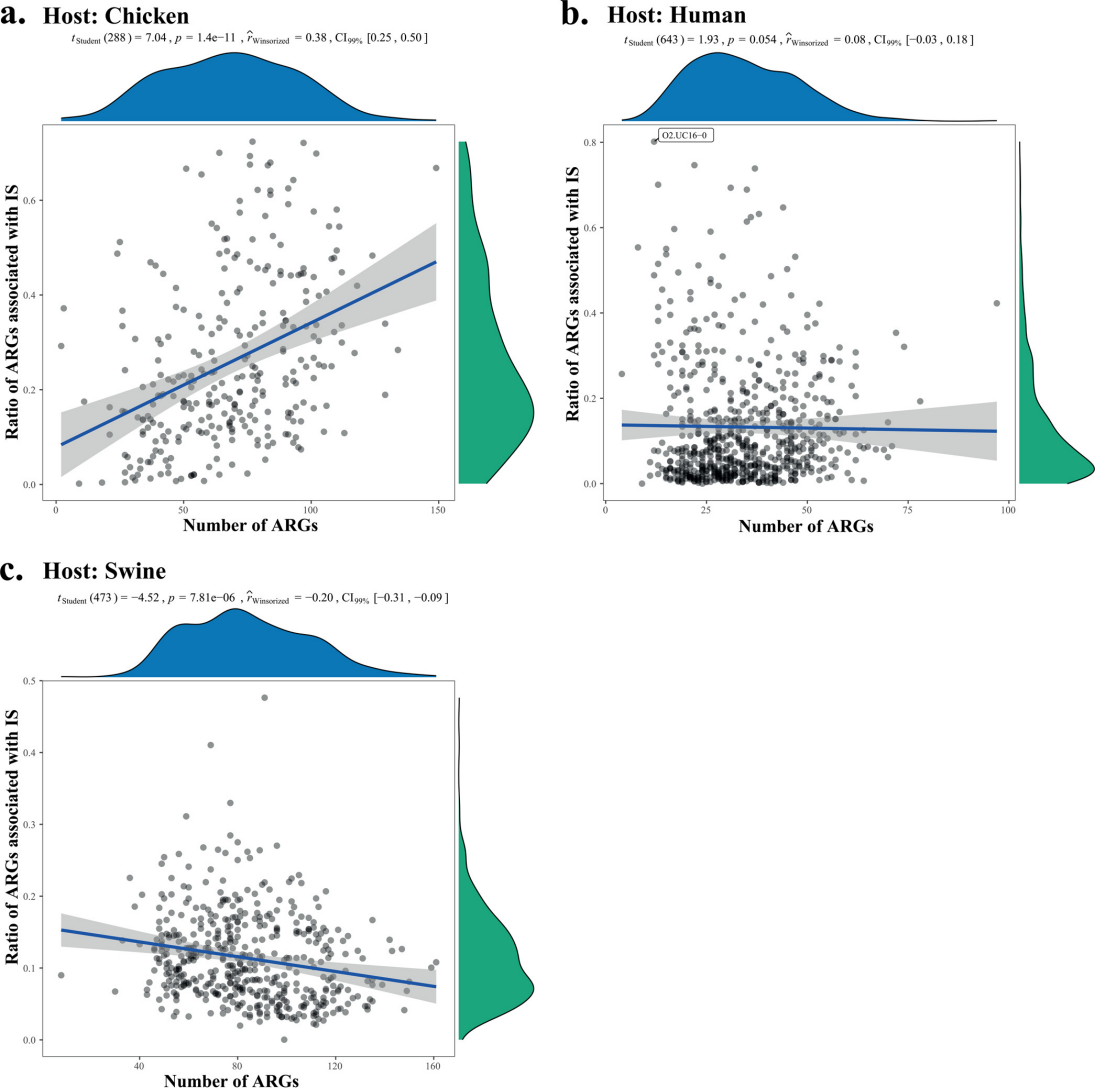
Model of the ARG transferability in different hosts. The transferability (*y* axis) was plotted with the number of ARGs in each sample (*x* axis). The transferability of ARGs in each sample is represented by the transmission index that was calculated from the ARGs associated with insertion elements compared with the total ARGs in each sample. The percentage bend correlation coefficient robust test was done for each type of hosts with confidence intervals at a 0.99 level.

In the present study, the transferability of ARGs in both types of hosts was evaluated using an index, namely, Antibiotic Resistance Gene Transferability Index (ARGTI) that was calculated from the ratio of ARGs associated with mobile genetic elements compared with total ARGs in individual samples (Fig. 5). Generally, the ARGTT in a human host (median, 0.0831) is lower than that in swine (median, 0.1051) and chicken (median, 0.2180). A higher variance of ARGTI in human is observed than that in swine, consistent with the higher beta-diversity of ARGs in human hosts. Along with the increase of observed ARGs in each sample, ARGTI is negatively correlated with the number of ARGs in humans and swine, compared with a positive correlation in chicken (Fig. 5).

**FIG 6:**
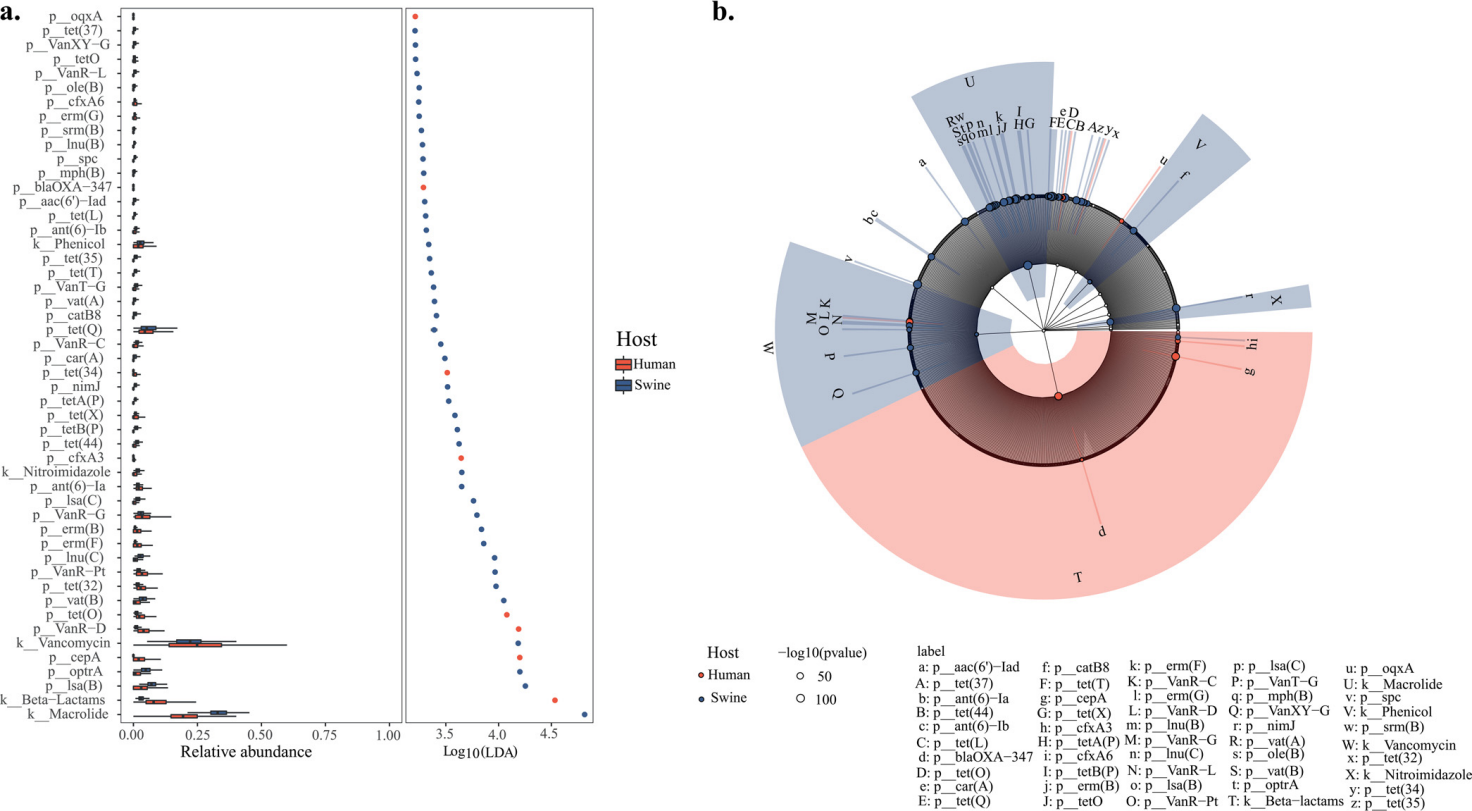
Linear discriminant analysis effect size (LEfSe) was used to identify discriminative ARGs between human and food animals. (a) Histograms of linear discriminant analysis (LDA) scores of the relative abundance of ARGs inhabiting two types of hosts. Key types of differently abundant ARGs were identified using the LEfSe algorithm in different hosts, and their relative abundance was presented accordingly. (b) Cladograms derived from LEfSe analysis of differential ARGs in both types of hosts. The central point denotes the root of the tree and expanded to each ring representing the next lower taxonomic level from antibiotic classes to ARGs. Each circle’s diameter represents the relative abundance of the ARGs.

Linear discriminant analysis effect size (LEfSe) was used to identify discriminative ARG types between human and swine hosts (Fig. 6) demonstrating that ARGs conferring resistance to macrolides and beta-lactams are the main contributors.

## DISCUSSION

One Health studies have been performed to survey the relatedness of bacterial isolates carrying ARGs in human, animal, and environmental commensals, e.g., for Klebsiella pneumoniae (12) and E. coli (13). The case studied extensively is about methicillin-resistant Staphylococcus aureus (MRSA) CC398 in humans and pigs (14, 15). High pig density leading to a significant increase of MRSA CC398 in hospitals in Spain was proposed recently (16). The abundance of genes conferring resistance to six classes of antibiotics together with class 1 integrase and the abundance of IS*6100*-type transposons were observed in one study on swine farms from China (17). However, there is not much convincing evidence to show the relationships between pathogens of E. coli from food animal to bloodstream infection in human based on whole-genome studies (13), particularly commensal bacteria. Indeed, it is a challenge to identify sources of AMR, and horizontal gene transfer appears to play a more significant role than clonal expansion in the spread of AMR. In the present study, ARGs associated with mobile genetic elements and their bacterial hosts were investigated comprehensively in human and food animal fecal metagenomes. Through comparisons between food animals, with swine and chicken as examples, and human hosts, particularly swine samples from China and human samples from China and Hong Kong, the predominantly shared ARGs were identified. Their associated mobile genetic elements and bacterial hosts were identified. These shared ARGs are important to human health and deserve to be studied further in depth with more human microbiota data. Therefore, we hypothesize that the strong effects of ARGs in swine on the human population are greater than we have expected thus far. However, a caveat of current metagenome data set, particularly regarding ARGs from the Hong Kong population that were constrained to one study on colorectal cancer, is that researchers should be cautious before drawing any solid conclusions (18). On another hand, the transferable capability of ARGs should be validated with more information on MGEs that are integrated with ARGs, although one index has been developed in the present study.

Commensal bacteria affiliated with two major phyla, namely, *Firmicutes* and *Bacteroides*, have been observed as inhabiting the most abundant microbial habitats in human and food animals, including swine (19, 20). In the present study, *Clostridiales*, including *Clostridioides* and *Roseburia* species carrying Tn*4451*/Tn*4453* with diverse ARGs, were observed to be shared by human and swine hosts in China. The mobilizable transposons Tn*4451*/Tn*4453* could be excised, which was mediated by the product of the *tnpX* gene which encodes a member of the large resolvase (11). The transposase TnpX was identified widely in human and swine gut microbiota. This report is the first one about contributions of Tn*4451*/Tn*4453* to antibiotic resistance in the wide type of human and food animal hosts. Noticeably, this type of transferable cassettes carrying ARGs was observed in several commensal opportunistic bacterial hosts, including *Clostridioides*, Campylobacter, and *Bacteroides*, indicating their potential transferability. Overall, from what we know of modern food production, it is clear that antibiotic use does contribute to the resistance of our commensal and pathogenic microflora, but the scale of this contribution has not yet been quantified. We find that ARGs are more likely to be spread by conjugation via integrative conjugative elements or integrative mobilizable elements than transduction via prophages. Particularly, a new multidrug resistance gene, *cfr(C)*, identified in recent studies was observed within this type of common transposon, namely, Tn*4451*/Tn*4453*, in commensal bacteria. The variable commensal bacterial hosts of this type of transposon suggest the potential transmission of this mechanism via the foodborne route, warranting enhanced efforts to monitor its spread in foodborne pathogens.

Within the human host, ARGs are diverse in populations from different regions with India having the most prominently high observations followed by the human cohort in Hong Kong and China. China and India have been reported as the hot pots of AMR in food animals (3). The link between swine and host in China and Hong Kong proposed in the study possibly might explain the high abundance of ARGs in India. Unfortunately, the resistome data in food animals in India is lacking currently. All these data suggest the importance of One Health to surveil AMR in humans and food animals together, although the similarity degree between swine and human hosts in different regions is different. On another hand, even in the same region, the heterogeneous compositions of ARGs were observed. However, there are no correlations between age and ARGs in the cohort from Hong Kong, and different age groups also possibly carry different types of relative abundances of ARGs, e.g., in the Hong Kong cohort. This information necessitates the development of personal and specific therapies to treat infections in people. Considering the diversity of ARGs, there is no association between ARGs and age, as even the abundance of ARGs in old people is higher than that in younger people. However, the dominant group of bacteria carrying ARGs associated with mobile genetic elements is similar within the Hong Kong cohort. Additionally, several types of ARGs were observed with an extraordinarily high abundance compared with contigs they were from in the Hong Kong cohort. All these data possibly show insights into the choice of antibiotics for therapy.

The main strengths of this study are that ARGs from diverse groups of the Hong Kong cohort were surveyed and mobile genetic elements correlated with the diversity of ARGs. Shared relationships between swine and humans in China were explored. Nonetheless, the study has several limitations. First, because of the weakness of the sequencing methods, namely, the short-read sequencing Illumina platform, we suppose that a high ratio of mobile genetic elements has been disrupted from associated ARGs, as shown in the whole-genome sequencing on culture pathogens, leading us to evaluate the transferable capability of ARGs. However, the low ratio of contigs possessing ISs was observed, and it also excludes the intrinsic existence of fewer ARGs located adjacent to mobile genetic elements, leading to less of an opportunity for them to transfer within human gut microbiota. Second, minor groups of bacteria have still not been studied completely. Third, sampling size was insufficient to make solid conclusions, as was shown in the rarefaction curves of the diversity of ARGs in both hosts. All these limitations might be resolved in the future with more efforts on samplings and sequencing depths with different sequencing platforms, including the Nanopore sequencer. Finally, the intestinal source locations (e.g., ileum, cecum, and colon) for the majority of the gut metagenomes were not available. Therefore, the ARGs could not be further analyzed by intestinal source locations, although it is recognized that the microbiome compositions of gut locations are different in terms of microbial abundance and diversity (21).

In conclusion, potential AMR hot spots were observed in the present study with a comprehensive surveillance of the resistome, especially those ARGs that are the most likely candidates for horizontal transfer to pathogens. This study emphasizes on the importance of surveillance for detecting emerging resistance threats before they spread, so as to mitigate threats. The knowledge from this study will benefit pathogen control and prevention efforts to protect human health.

## MATERIALS AND METHODS

### Sampling and sequencing.

In the present study, 21 subjects (all volunteers) who met the following inclusion criteria were recruited: (i) not currently suffering from any acute illness, including fever, acute gastroenteritis, or acute respiratory tract infection; (ii) not currently taking any antibiotics in the past 3 months; (iii) no intake of probiotic supplements; and (iv) no history of hospitalization in the past 3 months. In total, 11 male and 10 female subjects were recruited.

In addition, metagenomes (*n*** = **1,465) of slaughter pigs from European countries (*n* = 181) (22) and China (*n* = 295) (20) and poultries from China (*n* = 135) and European countries (*n* = 178), as well of humans from several regions (23–28), including one study on a Hong Kong population (18), were integrated with 21 newly sequenced metagenomes of a Hong Kong cohort and one swine from Hong Kong for further analyses of the resistome (Table 1 and Fig. 1).

A standardized fecal sampling protocol to process stool samples was followed in the present study (29). Sequencing was conducted on a NextSeq platform (Illumina, San Diego, CA) at Novogene Co., Ltd. (Beijing, China).

The study met the standards for the ethical treatment of participants and was approved by the Institutional Review Board of the University of Hong Kong/Hospital Authority Hong Kong West Cluster (reference number UW 18–278). Study participants gave informed consent.

### Metagenome assembling.

To be consistent, all samples used in the present study were processed using the same pipeline from quality control, gene assembly and gene annotations, to taxonomic study.

An in-house pipeline was developed to perform calling of ARG contigs assembled from raw reads in the present study. Briefly, quality control was performed using Trimmomatic (30). Host (human/swine) DNA removal was done by alignment against host genomes using BWA-MEM (31) and SAMtools (32). Reference genomes of the hosts are WTSI_X_Y_pig V2 (GCF_000003025.6) for swine and Hg19 (GCA_000001405.26) for human. The metagenome module of SPAdes was used to do assemble (33). Qualified reads were submitted to the Metagenomic Phylogenetic Analysis pipeline (MetaPhlAn2) for taxonomic profiling (34). Taxonomic identification on strain level was performed using StrainPhlAn (35). Kraken2 was also used to validate taxonomic analyses using contigs (36). MetaBAT2 (37) was used to bin genomes from contigs, and CheckM was performed to assess the quality of bins (38).

### ARG calling.

Genes was called using MetaGeneMark from contigs longer than 500 bp (39) and searched against the most recent version of the ARG databases of ResFinder (updated in September 2020) (40). A BLAST E value of 1e-10, a 70% query coverage, and an 80% similarity as suggested previously (23) were adopted to filter searching results using in-house python scripts.

For ARGs, the classification of antibiotics was inferred based on the WHO ATC code J01 (https://www.whocc.no/atc_ddd_index/). For each class of antibiotics, the sum of the relative abundance of each antibiotic resistance gene mapped to the same antibiotic was categorized for this type of antibiotics (23). All matrix files were generated using custom python and R scripts.

Rarefaction curves for ARGs in both hosts were extrapolated using iNEXT (41). Sørensen-based multiple-site dissimilarities in both types of hosts were calculated using R package betapart with 100 resample times for 10 samples (42). Shannon and Simpson diversities were calculated using R package vegan (https://cran.r-project.org/web/packages/vegan/). The number of present and shared ARGs was calculated for the compared sample types, and Venn diagrams were drawn in R using the draw.pairwise.venn command of the VennDiagram package (https://CRAN.R-project.org/package=VennDiagram). Negative binomial general linearized models (GLMs) were used to predict the means and standard errors (SEs) of the relative sum abundances in different sample types with Turkey’s *post hoc* test to correct since the distributions of ARGs do not fit a normal or Poisson distribution due to overdispersion. For principal coordinate analysis (PCoA), raw matrix files were used to generate the Bray-Curtis distance matrix that was submitted to the R package vegan. A permutational multivariate analysis of variance (PERMANOVA) between different groups was done with adonis in vegan with the similarity index using 9,999 permutations, and the resulting *P* values were corrected with the Benjamini and Hochberg procedure for multiple testing using the p.adjust command in *R*. All plots, bar charts, PCoAs, and heatmaps were generated using the ggplot2 package (https://ggplot2.tidyverse.org/).

### Mobile genetic elements.

Integrons were identified using IntegronFinder v.1.4 with the –local_max option (43). Integrative conjugative elements (ICEs) and integrative mobilizable elements (IMEs) were detected using MacSyFinder v.1.0.2 with CONJScan profiles (44). Integrases were called using the PFAM profile PF00589 for tyrosine recombinases and profiles PF00239 and PF07508 for serine recombinases (45). Insertion sequences (ISs) were detected using ISfinder and ISEScan on contigs (46). Prophages and viruses were identified using an in-house script with vpn to run PHASTER (47) and Virfinder (48), respectively. Plasmids were predicted using PlasFlow (49) and PLSDB (50). Reads were aligned using BWA-MEM to all genes predicted using MetaGeneMark. pileup.sh from the bbmap suite v37.25 was used to calculate reads per kilobase per million (RPKM) and the number of reads mapped to each gene (BBMap short read aligner, http://sourceforge.net/projects/bbmap).

### ARGs coordinated with MGEs and taxonomy.

The co-occurrence of ARGs and MGEs was surveyed through identifying coordinates (<10 kb) between ARGs and MGEs on the same contig using in-house python scripts. On another hand, the Bray-Curtis distance matrix of species, ARGs, and MGEs was calculated with the *vegdist* command in the vegan package and compared to observe possible correlations between taxonomy and the resistome and mobilome that occurred in at least half of all samples. Comparisons were done using Mantel’s test from vegan. Network analyses were conducted in R using the vegan, igraph (https://igraph.org/r/), and Hmisc packages. Associations of ARGs between bacterial hosts and mobile genetic elements were also visualized with the HiveR R package (https://CRAN.R-project.org/package=HiveR).

### Variations of ARGs.

In the present study, a list of clinically significant ARGs was selected for further analyses on variants. For each ARG, genes (length, >70% coverage) present in pairs of hosts in the same regions were extracted and aligned using MAFFT with the L-INS-i option (51). The phylogenetic tree for each alignment was built using IQ-TEE (52), and the associated features including the relative abundance of each gene, flanking IS element, whether or not on plasmid, host source and geographic region were aligned with the tree using Ggtree (53). All these genes were also clustered using PopPUNK (54) and visualized with Cytoscape (https://cytoscape.org).

### Modeling transferability of ARGs.

In the present study, the antibiotic resistance gene transferability index (ARGTI) was developed to evaluate the risk of spread of ARGs within and between hosts.

The ARGTI is

(1) $$\text{ARGTI}=\overset{n}{\underset{i=1}{\sum }}\frac{S_{a-\mathrm{mi}}}{S_{\mathrm{ai}}}$$

in which, *S_a-mi_* stands for the sum of the abundance of ARGs that are associated with mobile genetic elements, while *S_ai_* represents the sum of the abundance of ARGs in individual microbiota.

### Effectors on ARGs.

Linear discriminant analysis effect size (LEfSe) was used to identify discriminative ARG types between human and swine (55). The alpha value for the factorial Kruskal-Wallis test and the pairwise Wilcoxon rank-sum test is 0.01. Multiclass analysis was performed using the all-against-all comparison with 3.0 as the threshold on the linear discriminant analysis (LDA) score. The false-discovery rate (FDR) correction for *P* values of the discriminative ARGs was performed using the Benjamini-Hochberg method. Significance was determined by using the *q* value of 0.01.

### Data availability.

The DNA sequences from 22 fecal samples are deposited in the European Nucleotide Archive under the project accession number PRJNA701076. All data, summarized figures, tables, and codes used in the study have been deposited online at https://github.com/huiluocao/gut_resistome.
