# Supplementary figures and images for: Sharing of Antimicrobial Resistance Genes between Humans and Food Animals
Source: mSystems. 2022 Oct 11;7(6):e00775-22. doi: 10.1128/msystems.00775-22 (PMC9765467; doi:10.1128/msystems.00775-22)

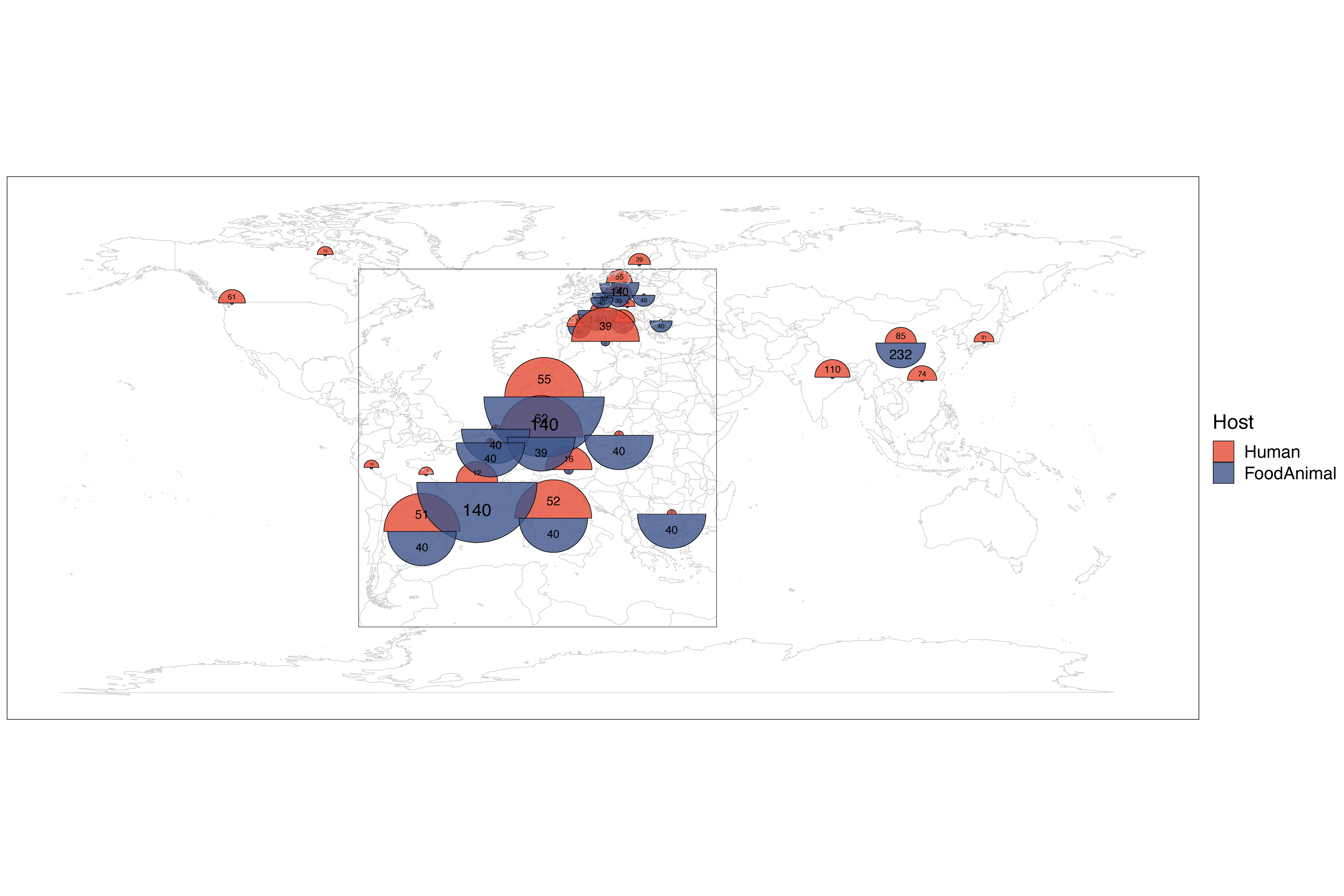

Supplement: FIG S1 [file msystems.00775-22-s0001.tif]

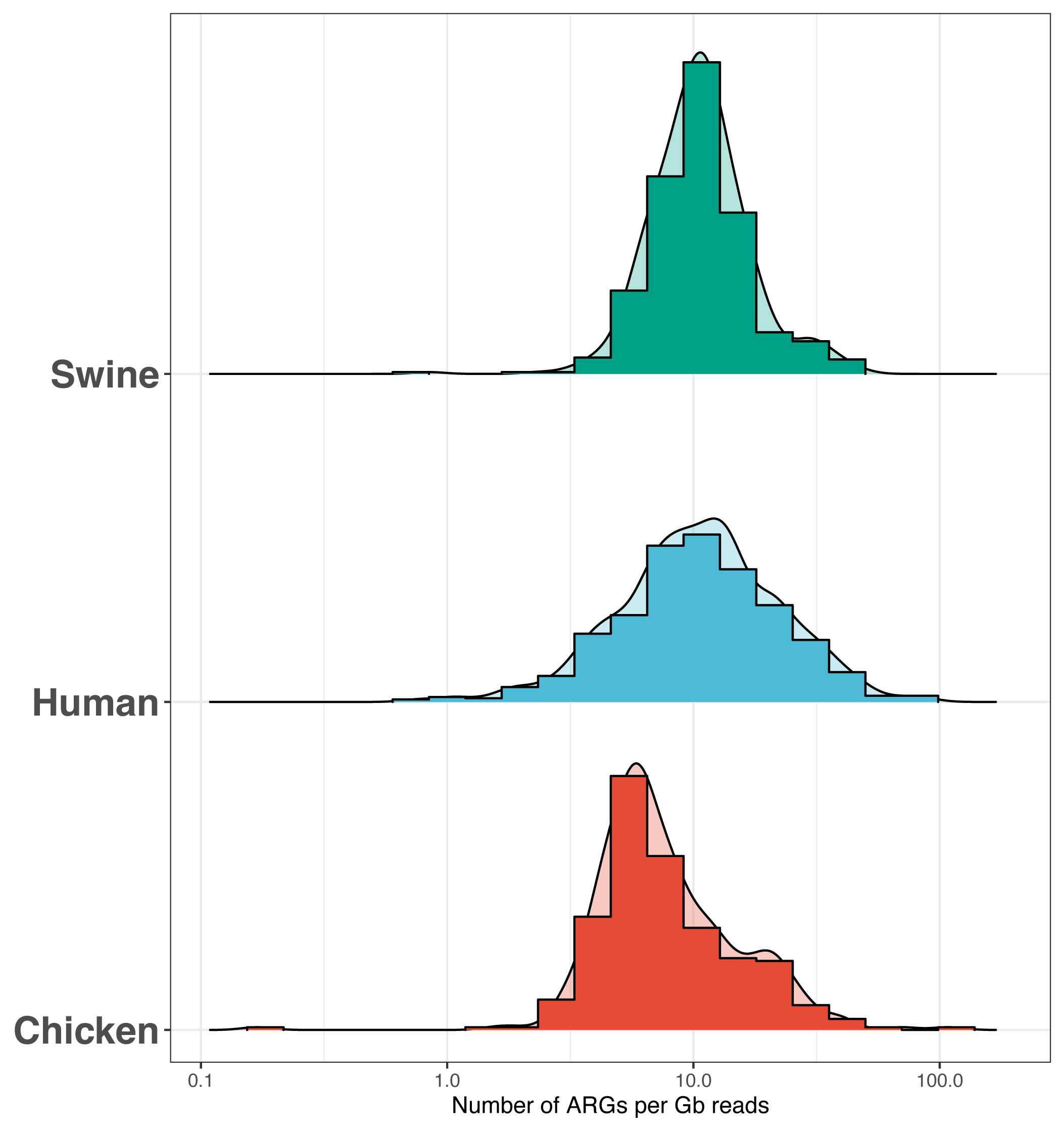

Supplement: FIG S2 [file msystems.00775-22-s0002.tif]

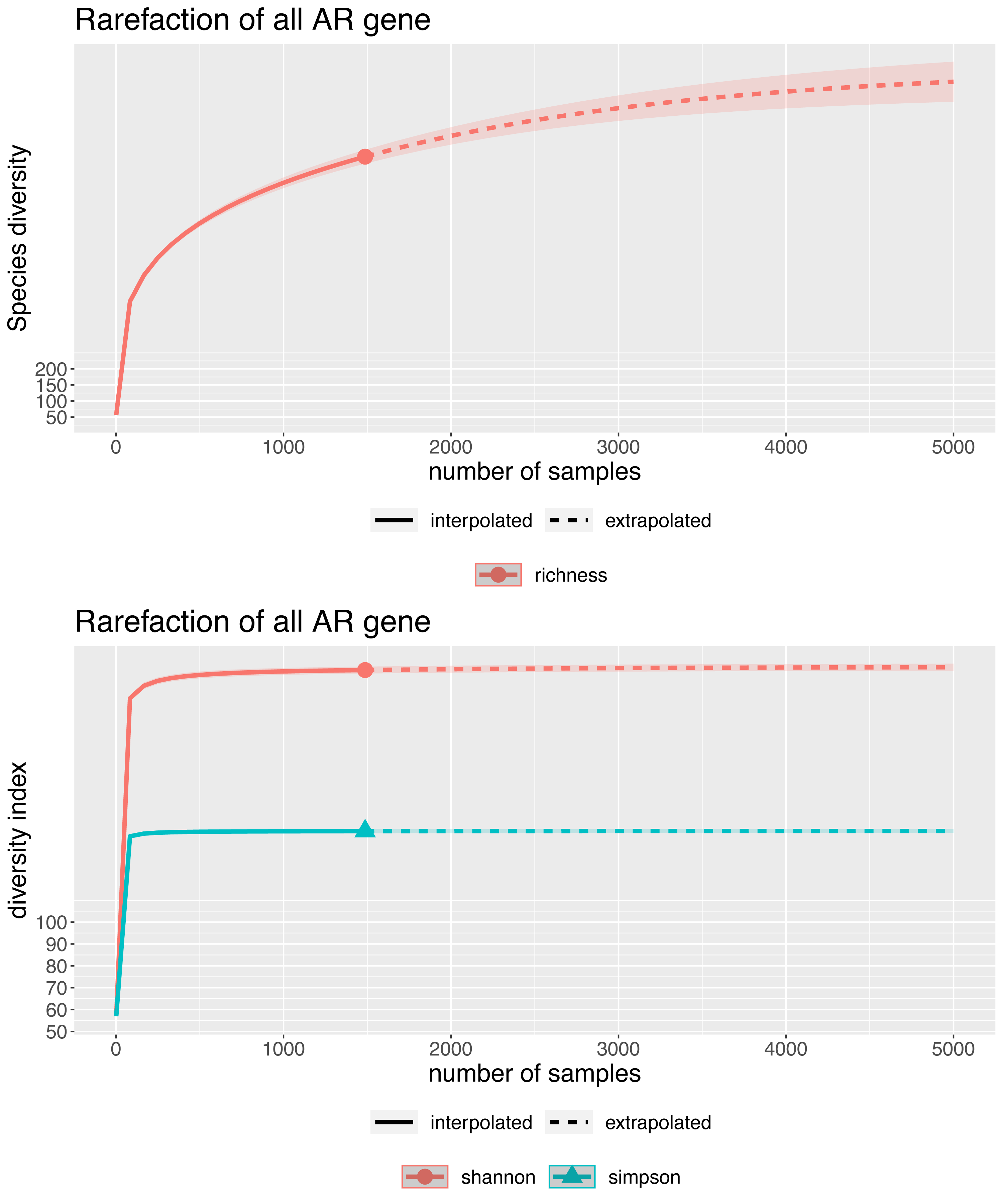

Supplement: FIG S3 [file msystems.00775-22-s0003.tif]

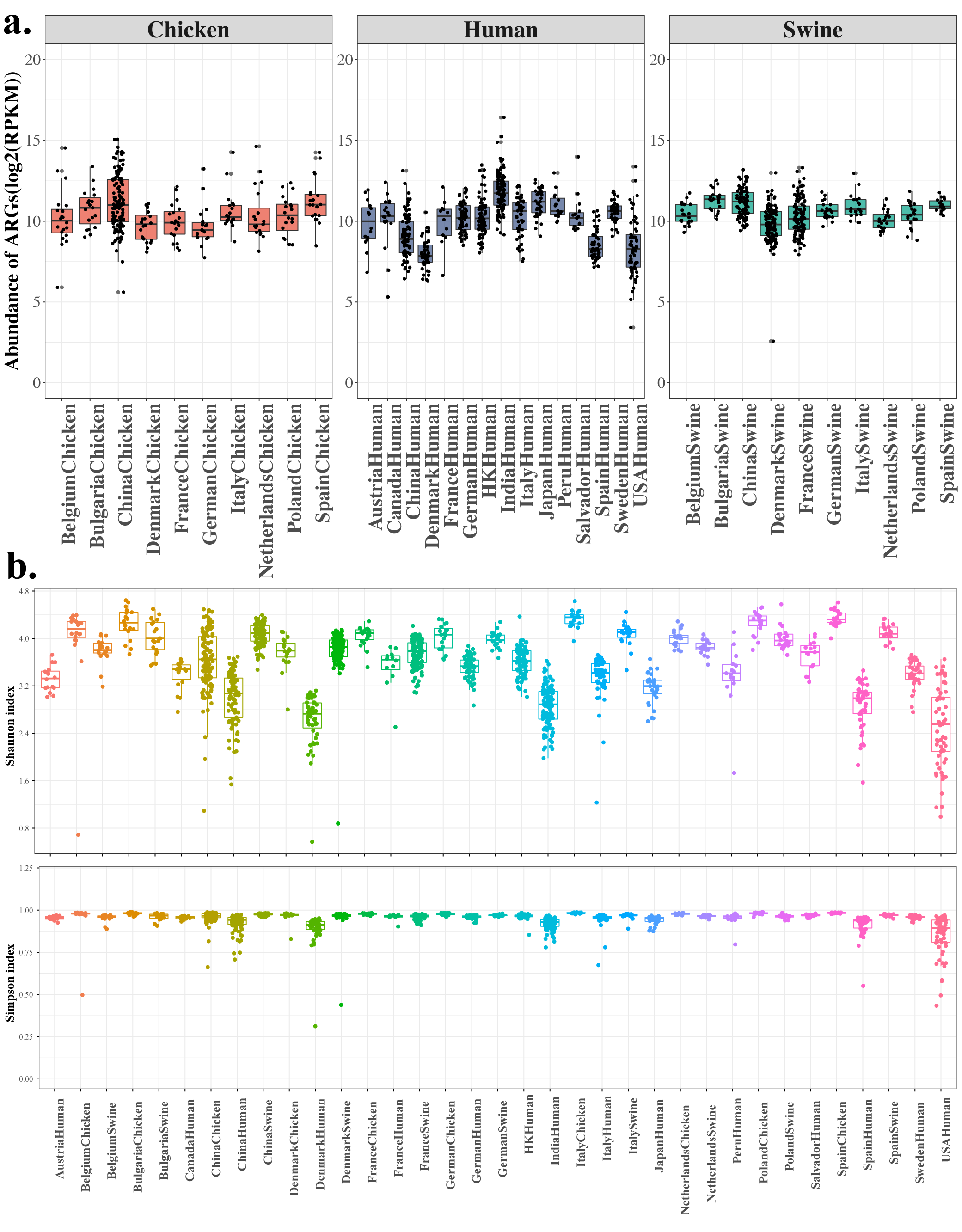

Supplement: FIG S4 [file msystems.00775-22-s0004.tif]

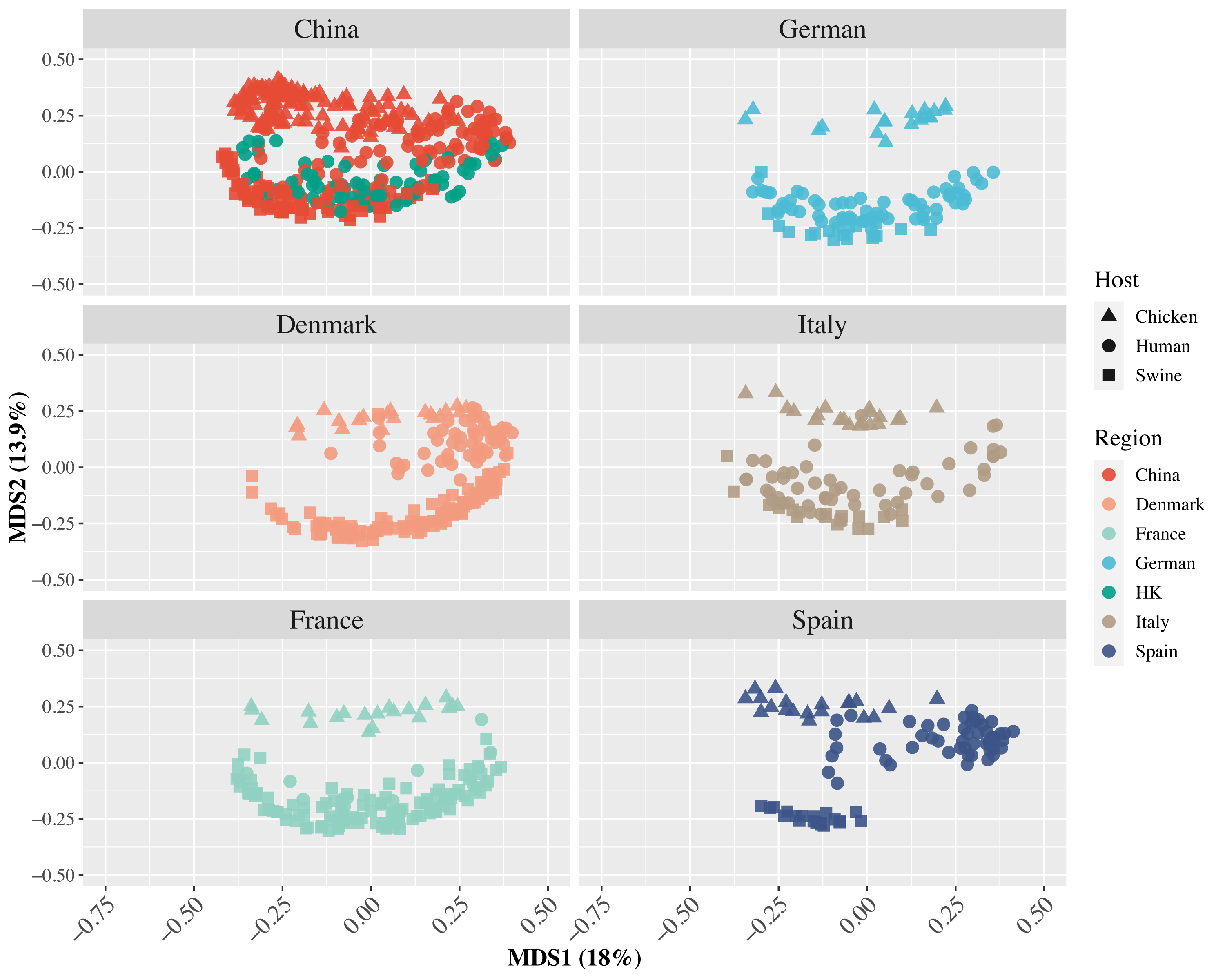

Supplement: FIG S5 [file msystems.00775-22-s0005.tif]

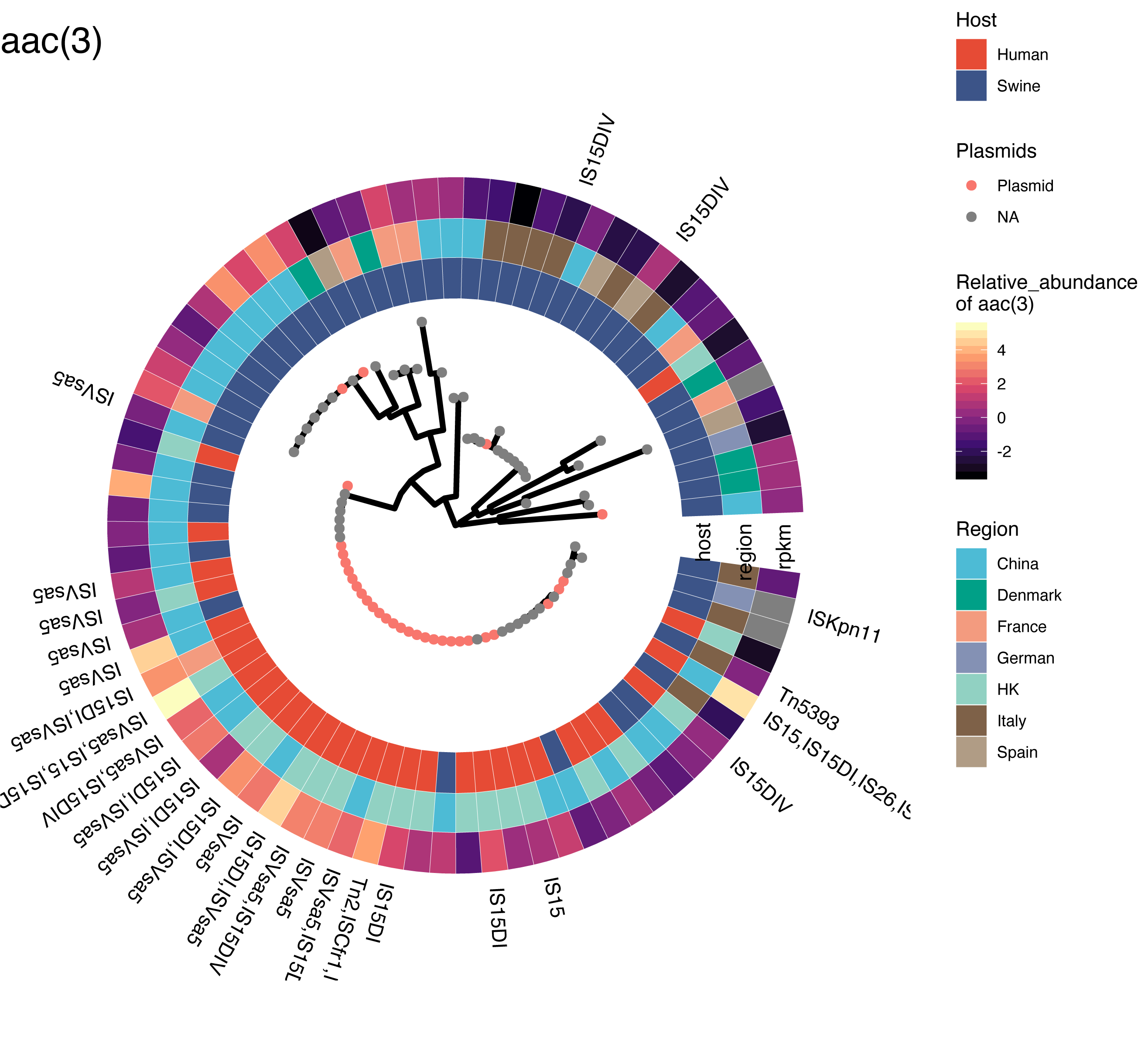

Supplement: FIG S6 [file msystems.00775-22-s0006.tif]

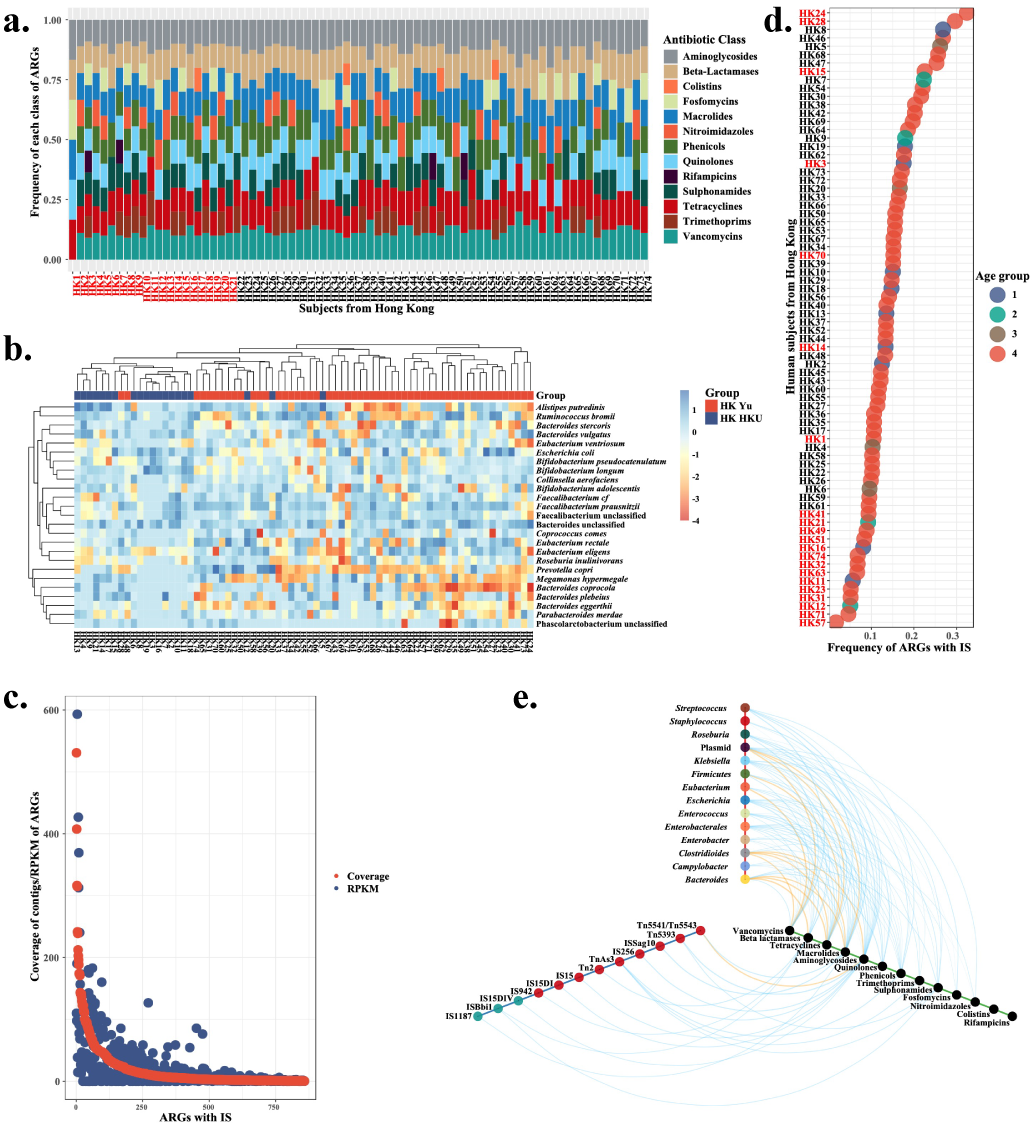

Supplement: FIG S7 [file msystems.00775-22-s0007.tif]
